# Amyloid‐beta (1–40) peptide is associated with systemic metabolic health

**DOI:** 10.1111/eci.70171

**Published:** 2026-01-08

**Authors:** Kateryna Sopova, Dimitrios Delialis, Evmorfia Aivalioti, Georgios Georgiopoulos, Stravros Athanasopoulos, Georgios Zervas, Christina Konstantaki, Marco Sachse, Martin Sigl, Daniel Duerschmied, Simon Tual‐Chalot, Kimon Stamatelopoulos, Konstantinos Stellos

**Affiliations:** ^1^ Department of Cardiology, Haemostaseology, and Medical Intensive Care University Medical Centre Mannheim, Medical Faculty Mannheim, Heidelberg University Mannheim Germany; ^2^ Department of Cardiovascular Research, European Center for Angioscience (ECAS), Medical Faculty Mannheim Heidelberg University Mannheim Germany; ^3^ German Centre for Cardiovascular Research (DZHK) Partner Site Heidelberg/Mannheim Mannheim Germany; ^4^ Department of Clinical Therapeutics National and Kapodistrian University of Athens Medical School Athens Greece; ^5^ Helmholtz‐Institute for Translational AngioCardioScience (HI‐TAC) of the Max Delbrück Center for Molecular Medicine in the Helmholtz Association (MDC) Heidelberg University Mannheim Germany; ^6^ Department of Medicine VI University Medical Centre Mannheim, Heidelberg University Mannheim Germany; ^7^ Biosciences Institute, Vascular Biology and Medicine Theme, Faculty of Medical Sciences Newcastle University Newcastle Upon Tyne UK

**Keywords:** amyloid‐beta, BARD score, cardiovascular disease, lipid metabolism, metabolic syndrome, triglyceride‐glucose index

## Abstract

**Background:**

Amyloid‐beta 1–40 peptide (Aβ40) has recently emerged as a blood‐based biomarker of cardiovascular disease (CVD). However, whether plasma levels of Aβ40 are associated with metabolic traits in humans without established CVD remains poorly understood.

**Methods:**

Aβ40 was measured in plasma by ELISA and metabolic traits (waist circumference, fasting triglycerides, fasting HDL cholesterol and fasting glucose) were determined in a general population (*n* = 449) of individuals who did not have clinically overt CVD. Triglyceride‐glucose index (TyG) was used to calculate the risk for insulin resistance. BARD score was used to calculate the risk for metabolic liver disease.

**Results:**

Aβ40 levels were associated with the presence of metabolic syndrome (OR: 1.41 95% CI: 1.13–1.76, *p* = .003), and with higher odds for increasing incidence of metabolic syndrome components, characterized by decreased HDL‐C levels (OR: 1.31 95% CI: 1.03–1.58, *p* = .017) and increased triglyceride levels (OR: 1.30 95% CI: 1.04–1.57, *p* = .033) after adjustment for traditional cardiovascular risk factors. Further, Aβ40 levels were associated with increased odds for TyG (OR: 1.26 95% CI: 1.03–1.57, *p* = .042) and increased odds for the presence of diabetes mellitus (OR: 1.35 95% CI: 1.04–1.76, *p* = .018) after adjustment for age and sex, smoking status, hypertension and dyslipidemia. Increased Aβ40 levels were associated with increased odds for BARD score ≥2 (OR: 1.41 95% CI: 1.04–2.04, *p* = .045) after adjustment for traditional cardiovascular risk factors.

**Conclusion:**

Our findings suggest that Aβ40 peptide is associated with metabolic traits and risk for metabolic disease. Future longitudinal studies are warranted to determine the prognostic value of Aβ40 for the development and progression of metabolic diseases.

## Graphical abstract

Plasma amyloid‐beta 1–40 (Aβ40) levels were measured in 449 individuals without overt cardiovascular disease. Higher Aβ40 was independently associated with metabolic syndrome, dyslipidemia (low HDL‐C, high triglycerides), insulin resistance (TyG index), diabetes mellitus and increased BARD score indicating liver fibrosis risk. These associations remained significant after adjustment for traditional risk factors. These findings suggest Aβ40 as a novel biomarker for early metabolic dysfunction and end‐organ damage. Future longitudinal studies should assess its prognostic value for metabolic disease progression.

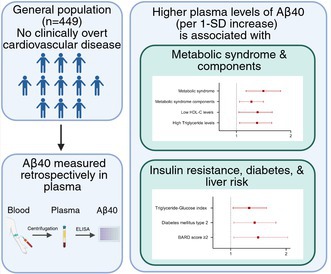

## 1 INTRODUCTION

Metabolic syndrome (MetS), characterized by a cluster of interrelated conditions including increased waist circumference, hypertension, elevated fasting triglycerides, low high‐density lipoprotein (HDL) cholesterol and elevated fasting glucose, is an important driver of cardiometabolic disease. MetS significantly increases the risk of type 2 diabetes, metabolic liver disease (metabolic dysfunction–associated steatotic liver disease, MASLD), cardiovascular disease (CVD), stroke or chronic kidney diseases, and premature death.^1^ The prevalence of MetS, driven mainly by overnutrition and sedentary lifestyle, varies from 12.5% to 31.4% worldwide,^2^ and its rising occurrence is now a major global public health concern.^3^ The development of MASLD, previously known as non‐alcoholic fatty liver disease (NAFLD), is tightly associated with MetS since 90% of the patients with NAFLD have more than one MetS component,^4^ while the risk of steatosis increases exponentially with each addition of components of the MetS.^5^ NAFLD covers a spectrum ranging from simple steatosis to steatohepatitis and/or liver fibrosis, and is associated with increased all‐cause and cardiovascular mortality.^6^, ^7^ In this context, identifying early biomarkers that can predict risk for metabolic dysfunction‐induced diseases in MetS patients is crucial.

Amyloid‐beta (Aβ) has recently emerged as a potential blood‐based biomarker associated with cardiovascular events and mortality.^8^, ^9^ Aβ is a proteolytic fragment of the amyloid precursor protein widely recognized for its central role in the pathogenesis of Alzheimer's disease (AD).^9^ β‐secretase (BACE1) is involved in APP cleavage, and further cutting by γ‐secretases generates peptides of length 40 (Aβ40, usually found in peripheral blood and vascular lesions) and 42 (Aβ42, usually found in AD‐associated brain lesions). Under normal conditions, an equilibrium exists between Aβ production and removal, while deregulation of this balance may lead to accumulation of Αβ in blood, vascular wall and heart tissues.^9^ Increased plasma Aβ40 has been associated with subclinical cardiac disease, as indicated by increased high‐sensitivity cardiac troponin T (hs‐cTnT), N‐terminal pro‐B‐type natriuretic peptide (NT‐proBNP) and lower left ventricular ejection fraction (LVEF).^10^ We have previously reported that Aβ40 blood levels are linked to the presence, extent and progression of carotid atherosclerosis in postmenopausal women,^11^ and associated with plaque composition and plaque burden in patients without clinically over atherosclerotic CVD.^12^ Additionally, circulating Aβ40 provides incremental prognostic value and enhances risk stratification in patients with stable coronary artery disease (CAD) and non‐ST elevation acute coronary syndrome for predicting new adverse cardiovascular events.^13^, ^14^ It is also independently associated with mortality in heart failure patients, probably due to a worsened cardiac function,^15^ while Aβ40 is associated with all‐cause mortality in patients at risk for atherosclerotic CVD partly mediated through renal dysfunction.^16^ Increasing evidence suggests that Aβ peptides may be linked to metabolic dysfunction. Circulating Aβ42 has been positively associated with BMI and blood pressure and inversely associated with HDL‐C levels, with higher serum Aβ42 concentrations observed in individuals with abdominal obesity, hypercholesterolemia, low HDL‐C and hypertension, all major features of MetS.^17^ Preclinical models demonstrated that Aβ42 increases in obesity are linked to vascular dysfunction and altered cardiac function.^18^, ^19^ Meanwhile, elevated circulating Aβ40 levels have been observed in patients with hypertension, diabetes mellitus, dyslipidemia or obesity.^20^, ^21^ Furthermore, the first line of therapy against these MetS components includes statins, anti‐hypertensives, angiotensin receptor inhibitors, anti‐thrombotics, anti‐diabetics or lifestyle medications.^22^ All these drugs may intervene in Aβ metabolism by modulating its production, secretion and clearance, affecting Aβ circulating levels.^8^ Together, these findings suggest that altered systemic Aβ metabolism may represent a link between metabolic dysregulation and vascular risk.

Nonetheless, despite this emerging evidence in established CVD, the clinical significance of Aβ40 in patients with MetS without overt CVD is scarce. The aim of our cohort study was to explore the clinical value of Aβ40 as an early metabolic blood‐based biomarker of metabolic dysfunction by investigating the association of plasma Aβ40 with metabolic traits and risk for metabolic disease in patients without established cardiovascular disease.

## 2 METHODS

### 2.1 Cohort

In this study, we performed a retrospectively designed post‐hoc analysis for a pool of consecutively recruited participants between 2015 and 2017 from the Athens Cardiometabolic Study. Recruitment was conducted at the Unit of Dyslipidemias and Atherosclerosis of the Department of Clinical Therapeutics, National and Kapodistrian University of Athens, as previously described.^12^ For this study, only patients without clinically overt ASCVD, resulting in a population of *n* = 888 patients. ASCVD was defined as: History of coronary artery disease (CAD) or history of ischemic stroke or transient ischemic attack or history of peripheral artery disease clinically overt or documented with imaging. From this group, Aβ40 was measured in plasma in a subgroup of *n* = 449 consecutively recruited patients between 12/2015 and 12/2017, based on sample size calculations as described in the statistical methods. Additional exclusion criteria included: withdrawal of consent, life expectancy <1‐year, severe valvular heart disease, myocarditis, end‐stage heart failure (New York Heart Association functional assessment, NYHA) IV or left ventricular ejection fraction <30%, end‐stage renal failure, active malignancy, autoimmune and infectious diseases. Medical history was obtained from all patients at baseline visit. The current study was conducted according to the principles of the Declaration of Helsinki. The Local Ethics Committee of Alexandra General Hospital approved the study's protocol (13/26.11.2015). Prior to enrollment, all study participants provided written informed consent.

### 2.2 Metabolic traits

We defined MetS as the presence of central obesity (waist circumference ≥94 cm for men and ≥80 cm for women) plus any two of the following, according to the International Diabetes Federation (IDF) criteria for European populations^23^: blood pressure ≥130/85 mmHg or the use of antihypertensive medication; plasma triglycerides ≥150 mg/dL or the use of lipid‐lowering medication; plasma HDL‐cholesterol levels <40 mg/dL for men and <50 mg/dL for women or the use of specific lipid‐modifying medication; hyperglycemia or prediabetes, defined as a fasting plasma glucose level ≥100 mg/dL or the use of specific antidiabetic medication. Hypertension was defined as office systolic blood pressure (SBP) ≥140 mmHg and/or diastolic blood pressure (DBP) ≥90 mmHg, confirmed by repeated measurements on at least two separate occasions, or current use of antihypertensive medication.^24^ Dyslipidemia was defined as LDL‐C levels exceeding the guideline‐recommended targets according to the ASCVD risk category determined by the SCORE2 and SCORE‐OP equations or by the presence of specific risk modifiers as outlined in current guidelines,^25^ or ongoing lipid‐lowering therapy. Diabetes mellitus type 2 was defined as fasting glucose >126 mg/dL in 2 successive measurements or HbA1C >6.5% or oral glucose tolerance test >200 mg/dL or use of antidiabetic medication.^26^ Finally, we implemented the triglyceride‐glucose index (TyG) as an additional measure of insulin resistance associated with adverse metabolic outcomes. BARD score is a serum‐based score that is commonly used to assess fibrosis severity of patients at risk for NAFLD. The BARD score considers diabetes mellitus type 2, BMI and AST/ALT ratio.

### 2.3 Biochemical measurements

Fasting blood samples were acquired with venipuncture. Plasma and serum samples were stored at −80°C until procession. Concentrations of Aβ40 in ethylenediaminetetraacetic acid (EDTA)‐plasma samples were measured in the baseline samples using a reliable enzyme‐linked immunosorbent assay (ELISA) kit manufactured by Biosource/Invitrogen in California, USA, as previously described.^13^ The intra‐ and inter‐assay coefficient of variance of the ELISA measurements in our laboratory was reported to be less than 8%. The minimum detectable concentration of human Aβ40 was <6 pg/mL (Biosource/Invitrogen, California, USA).

Fasting blood samples were collected for standard biochemical lipid profile, including total cholesterol, triglycerides, LDL‐C and high‐density lipoprotein cholesterol (HDL‐C) and high‐sensitivity C‐reactive protein (hs‐CRP). LDL‐C was measured using the Friedewald equation.

### 2.4 Statistical analysis

Baseline characteristics were summarized using means and percentages for continuous and categorical variables, respectively. Correlations between vascular and metabolic indices and Aβ40 levels were explored with Spearmans's correlation coefficients. Pairwise differences between Aβ40 tertiles were evaluated using the one‐way analysis of variance (ANOVA) for variables with normal distribution or the Kruskal–Wallis non‐parametric test for variables without normal distribution while the chi‐squared test was employed for categorical variables. Subsequently, we applied multivariable logistic regression analysis to examine the independent association of Aβ40 levels (i.e., per SD increase) with metabolic markers (i.e., presence vs. absence of components of MetS and MetS vs. non‐MetS). For all logistic regression analyses, binary outcomes were defined according to established clinical thresholds. Specifically, increased waist circumference was defined as ≥94 cm for men and ≥80 cm for women (IDF criteria for European populations)^23^; high triglycerides as ≥150 mg/dL or the use of lipid‐lowering medication; low HDL‐C as <40 mg/dL in men and <50 mg/dL in women or the use of lipid‐modifying medication; high blood pressure as systolic ≥130 mmHg and/or diastolic ≥85 mmHg or the use of antihypertensive medication; hyperglycemia/prediabetes as fasting plasma glucose ≥100 mg/dL or the use of antidiabetic medication; and TyG index in the highest tertile of its distribution. MetS was defined as the presence of central obesity (i.e., increased waist circumference) plus ≥2 of the above criteria according to the International Diabetes Federation (IDF) guidelines for European populations.^23^ The multivariable model was adjusted for a set of biologically plausible confounders, including age, sex, hypertension, dyslipidemia and smoking status (core model). Moreover, we implemented ordinal regression analysis to explore the association of Aβ40 levels with increasing MetS components after adjustment for the core model. The same multivariable model was implemented to examine the independent association of Aβ40 levels with increased risk for advanced fibrosis (BARD score ≥2). Regarding power considerations, the sample size of 449 observations provided an 85% power to detect a minimum increase of 50% (unadjusted) in the odds of presence of MetS per 1‐SD increase of Aβ40. Type I error was predefined at .05. All statistical analyses were performed using SPSS 21 (IBM Corporation, Armonk, New York). Statistical significance was defined at *p* < .05 unless stated otherwise. All tests were two‐tailed.

## 3 RESULTS

### 3.1 Population characteristics

Among *n* = 449 subjects without clinically overt cardiovascular disease, patients' Aβ40 levels at higher tertiles were older, with lower GFR and HDL‐C, higher SBP and TyG levels and higher prevalence of BARD score ≥2 (*p* < .05 for all) (Table 1). Patients with diabetes, hypertension, increased triglycerides and presence of MetS had higher mean levels of Aβ40 (*p* < .05 for all).

**TABLE 1:** Descriptive characteristics of the population by Aβ40 tertiles (*n* = 449).

	Lowest tertile (*n* = 149)	Middle tertile (*N* = 150)	Highest tertile (*N* = 150)	*p*‐Value
Sex [male], *n* (%)	60 (40.3)	67 (44.7)	72 (48.0)	.402
Age [years], mean (SD)	56.3 (11.4)	59.5 (11.9)	60.0 (12.2)	.012
Smoking, *n* (%)	48 (32.7)	53 (35.6)	42 (28.8)	.479
Hypertension, *n* (%)	56 (38.1)	65 (43.6)	69 (47.3)	.113
Dyslipidemia, *n* (%)	67 (45.60)	86 (57.7)	78 (53.3)	.159
Lipid‐lowering treatment, *n* (%)	37 (25.3)	53 (35.6)	46 (31.7)	.238
Diabetes mellitus type 2, *n* (%)	19 (12.9)	24 (16.10)	31 (21.2)	.158
BMI [kg/m^2^], mean (SD)	27.6 (5.1)	27.9 (3.9)	27.6 (4.5)	.887
GFR [mL/m^2^/min], mean (SD)	112.2 (40.6)	113.8 (45.7)	93.8 (40.1)	<.001
Total cholesterol [mg/dL], mean (SD)	205 (40)	201 (42)	201 (52)	.664
Triglycerides [mg/dL], median (IQR)	91 (69)	99 (65.5)	111 (79)	.055
LDL‐C [mg/dL], mean (SD)	128 (36)	124 (39)	127 (40)	.586
HDL‐C [mg/dL], mean (SD)	57 (15)	58 (18)	51 (16)	.005
SBP [mg/dL], mean (SD)	124.7 (16.5)	129.9 (19.6)	131.2 (21.3)	.01
DBP [mg/dL], mean (SD)	72.7 (10.9)	73.3 (10.1)	73.5 (11.1)	.775
Waist circumference [cm], mean (SD)	99.3 (14.1)	100.5 (9.9)	100.0 (13.4)	.724
TyG, mean (SD)	8.38 (.59)	8.50 (.56)	8.56 (.65)	.043
BARD score ≥2, *n* (%)	106 (77.4)	120 (83.9)	121 (90.3)	.015

*Note*: Continuous variables are presented as mean ± standard deviation (SD) for normally distributed data and as median (interquartile range, IQR) for non‐normally distributed data. *p*‐value is derived from parametric ANOVA test or nonparametric Kruskal–Wallis test or chi‐square test.

Abbreviations: BMI, Body mass index; DBP, Diastolic blood pressure; GFR, Glomerular filtration rate; HDL‐C, high‐density lipoprotein cholesterol; hs‐CRP, high‐sensitivity C‐reactive protein; LDL‐C, low‐density lipoprotein cholesterol; SBP, Systolic blood pressure.

### 3.2 Association between Aβ40 and systemic metabolic health

Increased Aβ40 levels were associated with decreased HDL‐C levels (OR: 1.31 95% CI: 1.03–1.58, *p* = .017 per 1‐SD increase), increased triglyceride levels (OR: 1.30 95% CI: 1.04–1.57, *p* = .033 per 1‐SD increase), increased odds for TyG in the highest tertile (OR: 1.26 95% CI: 1.03–1.57, *p* = .042 per 1‐SD increase) and increased odds for the presence of diabetes mellitus (OR: 1.35 95% CI: 1.04–1.76, *p* = .018) after adjustment for age and sex, smoking status, hypertension and dyslipidemia (Table 2). Aβ40 levels are associated with the presence of MetS (OR: 1.41 95% CI: 1.13–1.76, *p* = .003 per 1‐SD increase), and with higher odds for increasing incidence of MetS components (OR: 1.21 95% CI: 1.04–1.41, *p* = .007 per 1‐SD increase) and with the presence of BARD score ≥2 (OR: 1.41 95% CI: 1.04–2.04, *p* = .045) after multivariable adjustment (Table 2).

**TABLE 2:** Association of Aβ40 levels with metabolic traits.

	Univariate	Multivariable
	OR (95% CI)	
Prediabetes	1.35 (1.12–1.63) *p* = .02	1.16 (.93–1.41) *p* = .208
Diabetes mellitus type 2	1.52 (1.16–1.90) *p* = .001	1.35 (1.04–1.76) *p* = .017
High Blood pressure	1.26 (1.004–1.52) *p* = .025	1.12 (.93–1.41) *p* = .226
Low HDL‐C levels	1.30 (1.03–1.57) *p* = .013	1.31 (1.03–1.58) *p* = .017
High Triglyceride levels	1.26 (1.00–1.57) *p* = .033	1.30 (1.04–1.57) *p* = .033
Increased waist circumference	1.12 (1.02–1.52) *p* = .02	1.001 (.91–1.35) *p* = .347
TyG index	1.41 (1.12–1.69) *p* = .002	1.26 (1.03–1.57) *p* = .042
Metabolic syndrome components^a^	1.30 (1.08–1.52) *p* = .002	1.21 (1.04–1.41) *p* = .007
Metabolic syndrome	1.52 (1.21–1.90) *p* < .001	1.41 (1.13–1.76) *p* = .003
BARD score ≥2	1.46 (1.04–2.04) *p* = .023	1.41 (1.04–2.04) *p* = .045

*Note*: Prediabetes: fasting plasma glucose ≥100 mg/dL or use of antidiabetic medication, High blood pressure: blood pressure ≥130/85 mmHg or use of antihypertensive medication; High Triglyceride levels: triglycerides ≥150 mg/dL or use of lipid‐lowering medication; Low HDL‐C levels: HDL‐C <40 mg/dL in men and <50 mg/dL in women or use of specific lipid‐modifying medication, Increased waist circumference: waist circumference ≥94 cm for men and ≥80 cm for women, Metabolic syndrome was defined as central obesity plus two of the above mentioned criteria. OR represents the odds ratio for a patient with higher Aβ40 levels (per 1‐SD increase) to have deteriorated metabolic syndrome risk factors. Prediabetes, Low HDL‐C levels, High Triglyceride levels, increased waist circumference and TyG multivariable models are adjusted for sex, age, smoking status, hypertension and dyslipidemia. High blood pressure model was adjusted for sex, age, smoking status and dyslipidemia. Presence of metabolic syndrome are adjusted only for sex, age and smoking status to avoid collinearity with dyslipidemia.

Abbreviation: HDL‐C, high density lipoprotein cholesterol.

^a^ Estimates are derived from ordinal regression analysis for the increasing incidence of metabolic syndrome components per 1‐SD increase of Aβ40 after adjustment for age, sex and smoking status.

## 4 DISCUSSION

Our study investigated the relationships between Aβ40 levels and metabolic traits. Here, we provide the first evidence suggesting an association between Aβ40 levels and (1) an increasing incidence of MetS components, (2) the presence of MetS and (3) risk for metabolic disease defined as either high TyG or BARD score.

In the present study, we report that higher Aβ40 levels in patients were significantly associated with MetS components, particularly dyslipidemia, as characterized by lower HDL‐C levels and higher triglyceride levels after adjusting for potential confounders. Low HDL‐C and elevated triglyceride levels are essential components of MetS. Both are independently linked to a higher risk of coronary heart disease and have been targeted in clinical trials aimed at reducing cardiovascular events.^27^, ^28^ Decreased HDL‐c levels is an important risk factors for Aβ and cerebral small vessel disease burdens,^29^ while in cognitively normal individuals, increased levels of triglycerides at midlife predict increase of Aβ accumulation in the brain 20 years later.^30^ In transgenic AD mouse models, increased plasma triglyceride levels occur prior to the onset of Aβ deposition, but depend on Aβ presence in the circulation and reflect Aβ‐induced changes on peripheral lipid metabolism independently of inflammatory processes.^31^ The amyloidogenic pathway, the cleavage of APP by γ‐ and β‐secretases, occurs in lipid rafts, the membrane microdomains enriched in cholesterol and sphingolipids which both enhanced the production of Aβ. Indeed, the lipids may influence the membrane fluidity and affect secretases activity or Aβ aggregation kinetics.^32^ Nonetheless, this interaction goes both ways as both Aβ40 and Aβ42 can reduce sphingomyelin and cholesterol levels.^33^ Under pathophysiological conditions, Aβ40 and Aβ42 aggregate into oligomers or arrange into symmetric, periodic fibrils,^34^ while HDL particles interact with Aβ to prevent its aggregation into amyloid.^35^ These interactions can perturb membrane‐bound enzymes and receptors involved in lipid metabolism, such as ATP‐binding cassette transporters (ABCA1 and ABCG1) and the low‐density lipoprotein receptor (LDLR).^36^ ABCA1 and ABCG1 are crucial for the efflux of cholesterol and phospholipids to apolipoprotein A‐I (apoA‐I) and HDL particles, processes essential for maintaining cellular lipid homeostasis and preventing lipid accumulation. Dysregulation of these transporters by Aβ can lead to impaired cholesterol efflux, contributing to intracellular lipid accumulation and subsequent cellular dysfunction.^37^ Receptors of the low‐density lipoprotein receptor family, such as LRP1, are also tightly connected with APP metabolism as they participate in the production and clearance of Aβ.^38^ Furthermore, Aβ has been shown to interact with apolipoproteins, which are crucial for lipid transport and metabolism. Indeed, apolipoprotein J (ApoJ) and apolipoprotein E (ApoE) promote Aβ clearance with isoforms having differential effects on Aβ aggregation and degradation.^39^ The link between Aβ and lipid metabolism is reinforced by genome‐wide association studies showing that plasma Aβ levels are driven by genetic variants near ApoE locus.^40^ Further preclinical evidence also indicates that Aβ production and release into the bloodstream is also closely linked to lipid metabolism. For example, mice fed a saturated fats diet in exhibited a marked rise in Aβ synthesis in intestinal cells, associated with increased levels of circulating non‐esterified fatty acids and Aβ in the blood.^41^ Additionally, lipid‐lowering therapy in these mice led to a significant decrease in plasma Aβ levels.^42^ Collectively, this evidence suggests that Aβ's effects on lipid metabolism are multifaceted, involving direct interactions with lipid membranes, modulation of lipid transporters and activation of inflammatory pathways.

Higher Aβ40 levels were associated with risk for metabolic disease. Not only were Aβ40 levels independently associated with the presence of diabetes, but also increased Aβ40 levels were associated with increased odds for TyG, a surrogate marker of insulin resistance. The TyG index is a composite measure that integrates fasting blood glucose and triglycerides and high TyG index has been found associated with increased plasma Aβ40 in a non‐demented population.^43^ Further, we found a significant association between elevated Aβ40 levels and metabolic liver disease, as indicated by the association with the surrogate marker of advanced hepatic fibrosis, the BARD score. While the exact mechanisms underlying the relationship between Aβ40 and hepatic fibrosis remain unclear, our findings suggest that Aβ40 may be involved in hepatic fibrogenesis or serve as a marker of systemic metabolic disturbances that promote liver damage. The liver is one of the primary organs for peripheral Aβ clearance, with hepatocytes the major cell type of the liver responsible for Aβ clearance. Epidemiologic study has also demonstrated a significant association between liver function and cerebral Aβ accumulation.^44^ It has been recently proposed that MASLD was linked to elevated plasma Aβ levels in rats and humans, through compromised LRP‐1‐mediated peripheral Aβ clearance, while the carrier of the LRP‐1 rs1799986 T polymorphism showed marked reduction in Aβ levels.^45^ The connection between Aβ and MASLD is further supported by animal studies. Mice with MASLD exhibit increased Aβ plaque burden in the brain,^46^ while targeting Aβ clearance through the anti‐Aβ antibody NP106 ameliorated the extent of fatty liver and liver fibrosis.^47^ Therefore, Aβ40 may be involved in hepatic fibrogenesis or serve as a marker of systemic metabolic disturbances that promote liver damage.

Our findings align with and extend previous observations linking systemic Aβ metabolism to metabolic phenotypes. Serum Aβ42 is positively associated with components of MetS, higher BMI and diastolic blood pressure, and inversely with HDL‐C, and shows no association with triglycerides or fasting glucose.^17^, ^48^, ^49^ Our results now demonstrate that Aβ40 is associated not only with individual metabolic traits (i.e., high triglycerides and low HDL‐C), but also with the presence and burden of MetS components, and with surrogates of insulin resistance (TyG) and hepatic fibrosis risk (BARD) in individuals without overt CVD, which suggests a distinct role for Aβ40 in metabolic dysfunction. Our findings echo previous studies showing that 12 weeks of caloric restriction in overweight and obese individuals lead to reduced Aβ40 plasma levels.^50^ Considering that caloric restriction decreases plasma triglycerides and normalizes lipid profiles, Aβ40 modulation may occur through improved lipid metabolism. Recent translational studies outline potential mechanistic pathways connecting metabolic dysfunction and Aβ accumulation. Kallistatin, a serine proteinase inhibitor increased in patients with obesity, prediabetes and diabetes,^51^, ^52^ was shown to modulate systemic metabolism and promote amyloid plaque deposition and tau phosphorylation, thus linking metabolic disorders with neurodegenerative processes.^53^ Given that MetS biomarkers are associated with cognitive impairment and progression to dementia,^54^, ^55^ integrating plasma Aβ markers with neurostructural assessment to enhance early disease staging in ageing populations^56^ may enable longitudinal monitoring and earlier preventive programmes. Collectively, these findings support our hypothesis that Aβ40 may serve as a novel biomarker of systemic metabolic health.

Our study also has some limitations that should be considered. First, the cross‐sectional design limits our ability to infer causal relationships between Aβ40 and the observed metabolic traits. Longitudinal studies are needed to determine whether elevated Aβ40 levels predict the progression of MetS or hepatic fibrosis over time. Additionally, while we adjusted for multiple confounders, residual confounding from other factors cannot be excluded. Finally, our study population consisted of individuals without overt CVD, which may limit the generalisability of our findings to populations with established cardiovascular conditions.

Overall, our data suggest that Aβ40 may be a novel biomarker of systemic metabolic health. Increased Aβ40 plasma levels may help clinicians identify individuals at higher risk for developing MetS and its end‐organ damage, particularly those with dyslipidemia and elevated triglyceride levels, conditions that predispose to cardiovascular events.

## AUTHOR CONTRIBUTIONS

KSo, DD, EA, GG, SA, GZ, CK and MS were involved in data collection, formal analysis and methodology. KSo, DD, GG, KSta and KSte were involved in data curation. KSo drafted the manuscript with input from all coauthors; MS, IA, DD and STC provided conceptual advice and contributed to data interpretation. DD and KSte were involved in funding acquisition and resources. Each author contributed important intellectual content during manuscript drafting or revision and agrees to be personally accountable for the individual's own contributions and to ensure that questions pertaining to the accuracy or integrity of any portion of the work, even one in which the author was not directly involved, are appropriately investigated and resolved, including with documentation in the literature if appropriate.

## FUNDING INFORMATION

KSo is supported by the Program for Promoting Gender Equality and the Careers of Female Physicians and Scientists at University Medical Centre Mannheim, Medical Faculty Mannheim, Heidelberg University. ST‐C is supported by the British Heart Foundation (PG/23/11093) and the Royal Society (RG\R1\241197). DD and KSte are supported by the German Research Foundation Deutsche Forschungsgemeinschaft (DFG) (CRC1366 C07, project no. 394046768) and the Helmholtz Institute for Translational AngioCardioScience (HI‐TAC) of the Max Delbrück Center for Molecular Medicine in the Helmholtz Association (MDC), Heidelberg University, Mannheim, Germany.

## CONFLICT OF INTEREST STATEMENT

The authors declare that the research was conducted in the absence of any commercial or financial relationships that could be construed as a potential conflict of interest.

## Data Availability

Anonymized data can be requested after publication of the results of prespecified analyses from the corresponding authors to be shared subject to approval of institutional review boards.
